# Various Indicators for the Assessment of Hospitals' Performance Status: Differences and Similarities

**DOI:** 10.5812/ircmj.12950

**Published:** 2014-04-05

**Authors:** Farhad Lotfi, Rohollah Kalhor, Peivand Bastani, Nasrin Shaarbafchi Zadeh, Maryam Eslamian, Mohammad Reza Dehghani, Mohamad Zakaria Kiaee

**Affiliations:** 1 School of Health Management and Information Sciences, Iran University of Medical Sciences, Tehran, IR Iran; 2 Research Center for Health Information Management, Hormozgan University of Medical Sciences, Bandar Abbas, IR Iran; 3 Department of Health Service Management, School of Public Health, Qazvin University of Medical Sciences, Qazvin, IR Iran; 4 School of Health Management and Information Sciences, Shiraz University of Medical Sciences, Shiraz, IR Iran; 5 Health Management and Economics Research Center, School of Health Management and Information Sciences, Iran University of Medical Sciences, Tehran, IR Iran; 6 Research Center for Health Services Management, Institute of Futures Studies in Health, School of Management and Medical Information Science, Kerman University of Medical Sciences, Kerman, IR Iran; 7 Treatment Deputy, Ahvaz Jundishapur University of Medical Sciences, Ahvaz, IR Iran; 8 Education Development Center, Shiraz University of Medical Sciences, Shiraz, IR Iran

**Keywords:** Pabon Lasso, DEA, Malmquist Index, Hospital Performance

## Abstract

**Background:**

Hospitals are the most costly operational and really important units of health system because they consume about 50%-89% of total health resources. Therefore efficient use of resources could help in saving and reallocating the financial and physical resources.

**Objectives:**

The aim of this study was to obtain an overview of hospitals' performance status by applying different techniques, to compare similarities and differences between these methods and suggest the most comprehensive and practical method of appraisal for managers and policy makers.

**Patients and Methods:**

This is a cross sectional study conducted in all hospitals of Ahvaz (eight hospitals affiliated with Jundishapur University of Medical Sciences and eight non-affiliated hospitals) during 2007 to 2011. Two kinds of data were collected through separate special checklists. Excel 2007 and Windeap 2.1 software were applied for data analysis.

**Results:**

The present findings show that the average of bed occupancy rate (BOR) in the studied hospitals was about 65.91 ± 1.16. The maximum number of inefficient hospitals in the present study happened in the years 2007, 2008 and 2010 (four hospitals) but there were two hospitals in the third part of the present graph which had maximum level of efficiency and optimal level of productivity in the years 2007 and 2009. Data Envelopment Analysis (DEA) showed that the mean score of technical efficiency for the studied hospitals is 0.924 ± 0.105 with the minimum of 0.585 ± 0.905 for hospital number 1. Furthermore It shows that only five hospitals (31.25%) reach complete technical efficiency (TE) scores across all five years of 2007-11 (TE = 1).

**Conclusions:**

Results of the present and similar studies should be considered for the future planning and resource allocation of Iranian public hospitals. At the same time it is very important to consider need assessment results for each region according to its potentials, population under the coverage and other geographical and cultural indices. Furthermore because of potential limitations of each of the above models it is highly recommended to apply different methods of performance evaluation to reach a complete and real status view of the hospitals for future planning.

## 1. Background

Health is an absolute right of the all members of the society and health care sector is considered as one of the most significant service sections, so its indicators are considered as one of the main criteria developmental level and social welfare of any country (1). On the other hand, health sector resources are one of the issues addressed in most developing countries because more than five percent of gross domestic products (GDP) and about five to 10 percent of government's expenditure is usually allocated to this sector (2). So, as health care costs consume about 5%-10% of GDP in the most underdeveloped countries such as Iran, all the institutions providing health services including hospitals will be facing budget issues in the future (3).

Hospitals are the most costly operational and really important units of health system because they consume about 50%-89% of total health resources (4). National statistics show that about 40% of governmental health expenses are related to hospital care in Iran (5). Hospitals, especially in developing countries are recognized by non-efficient management of resources, low profitability, providing non-friendly and non-professional services, non-reflective hierarchial organizational structure and lack of function-based perks. Such a weak management of hospitals may lead to wasting of the resources (6). Decreasing these barriers in “efficiency” has been proposed as a useful indicator for hospital's performance assessment (7) which is expressed as the percentage calculated as the ratio of the total output power to total input power under specified conditions (8). This indicator for a hospital means that how this unit is successful in producing maximal output for a given set of inputs or how they generate the constant amount of output using minimal resources (7). There are different techniques assessing this indicator including hospital performance ratios, Data Envelopment Analysis (DEA), stochastic frontier analysis (SFA), Pabon lasso and so on.

Ratio analysis as one of the non parametric technical-efficiency assessment methods, which includes the separate examination of various key measures such as average cost per inpatient day, bed occupancy rate, average length of stay, bed turnover ratio and so on (9). But as it is very difficult to justify hospitals' performances applying many separate indicators (ratios), three of the most important are merged as Pabon lasso model. This graphical model uses three indicators to evaluate the overall performance of a hospital including: bed occupancy rate (BOR), bed turn over (BTO) and average length of stay (ALS) (10).

SFA is a parametric linear programming method that uses an integrated data set considering a special form of production function for assessing efficiency but because of some potential challenges, it is suggested to be used along with other techniques (11). DEA, a non-parametric linear programming method, was introduced as a superior method in a report by World Health Organization in 2003 for measuring efficiency in health systems (12). And finally, productivity is another mixed index using Malmquist index as a combination of distance function and DEA indicating total productivity of a Decision Making Unit like hospital (13).

## 2. Objectives

This study was conducted to present an overview of the efficiency of all hospitals in Ahvaz (affiliated and nonaffiliated with Ahvaz University of Medical Sciences) applying Pabon Lasso, DEA and Malmquist indicators to compare similarities and differences between the results obtained by these methods and suggest the most comprehensive and practical ones for managers and policy makers.

## 3. Patients and Methods

This cross sectional study was conducted in all 16 hospitals of Ahvaz (eight affiliated and eight nonaffiliated with Ahvaz Jundishapur University of Medical Sciences), Ahvaz, Iran during 2007 to 2011. All of them were specialized hospitals. Hospitals No. 1-8 were affiliated with the Jundishapur University of Medical Sciences in Ahvaz and the rest were nonaffiliated. Two kinds of data were collected through separate special checklists, one for indicators of Pabon Lasso and the other for assessing efficiency according to DEA and both were designed based on the research goals. Pabon lasso is a graphical model for demonstrating hospital performance using three indicators: bed occupancy rate (BOR), bed turnover (BTO) and average length of stay (ALS). This graph compartmentalizes the hospitals into four divisions: The first part stands for those hospitals with low BTO and BOR implying a surplus in hospital beds against the existing demand, however the second sector shows the hospitals with high BTO and low BOR which indicates unnecessary hospitalizations, an oversupply of beds, or using the hospital beds for simply observing patients. The third segment indicates those hospitals with high BTO and BOR that simply means an appropriate level of efficiency, with relatively few vacant beds at any time and finally the last category presents the low BTO and high BOR that may emerge because of admitting patients with chronic diseases or unnecessarily long ALS (7).

The first checklist included the number of active beds, number of active bed-days, number of occupied bed-days, number of discharges and performance data consisting bed occupancy rate (BOR), bed turn over (BTO), and average length of stay (ALS). The items of these checklists were filled for each of the studied years. Data achieved from the first checklists, was analyzed using Excel 2007 according to Pabon lasso model for presenting the related graphs. Then, another checklist was designed for obtaining the necessary data in order to form an input – oriented DEA model. Variables were categorized as inputs (number of physicians, number of nurses, number of other personnel and number of active beds) and bed occupancy rate number of patients and number of operations were considered as outputs. The related data were collected from the statistic's center of the Jundishapur University of Medical Sciences and other hospitals for a total of 16 hospitals during 2007-2011. Windeap 2.1 software was applied for further analysis.

## 4. Results

**Figure 1.**
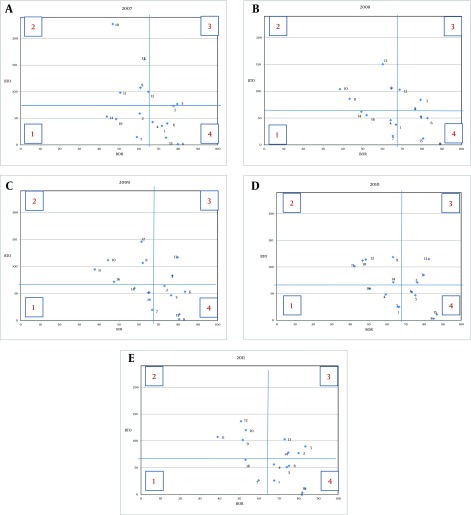
Pabon Lasso Graph Indicating the Performance Status of Ahvaz Hospitals in 2007-2011

Three determinant indicators constructing Pabon Lasso model are presented in Table 1. As this table indicates, the maximum rate of Bed Occupancy Rate (BOR) belonged to the year 2011 (66.71 ± 13.81) versus the minimum rate for the year 2007 (63.86 ± 11.88). Bed turn over (BTO) was in the highest range in the year 2007 (72.02 ± 58.18) against the year 2008 with the lowest rate (64.57 ± 39.46). The third factor was average long of stay (ALS) with the maximum of 17.17 days for the year 2007 versus 10.59 days in 2011 (Table 1). Figure 1 indicates the differences among the studied hospitals' status during 2007-2011. As Pabon Lasso graph implies, there were four hospitals in 2007, 2008 and 2010 in the first zone that represent inefficiency and underuse of resources in these units, this number decreased to three hospitals in 2009 and two hospitals in 2011.

**Table 1.** Determinant Ratios for Pabon Lasso Model in the Studied Hospitals During 2007-2011 ^a^

Year	BOR	BTO	ALS
**2007**	63.86 ± 11.88	72.02 ± 58.18	17.17
**2008**	66.27 ± 14.87	64.57 ± 39.46	15.12
**2009**	66.18 ± 13.93	67.36 ± 40.42	13.21
**2010**	66.51 ± 14.27	66.26 ± 38.67	11.93
**2011**	66.71 ± 13.81	69.10 ± 39.64	10.59
**Mean ± SD**	65.91 ± 1.16	67.86 ± 2.85	13.60 ± 2.60

^a^ Data are presented in Mean ± SD.

In contrast, there were only two hospitals in the third part of the graph which stands for efficiency and optimal level of productivity in the years 2007 and 2009. This number has been increased to three hospitals in 2008 and 2010, and 4 hospitals in 2011 as fully efficient units. Another important finding of this graph is that most of the hospitals were located in the second and the forth zone during 2007 to 2011 which may be due to unnecessary hospitalizations, an oversupply of beds, using the hospital beds for simply observing patients (second zone) or admitting chronic patients with unnecessarily long ALS (forth zone)(Figure 1). The average score of hospitals' technical efficiency is presented in Table 2. This table indicates that the mean score of technical efficiency for the studied hospitals is 0.924 ± 0.105 with the minimum of 0.585 ± 0.905 for hospital number 1. Furthermore It shows that only five hospitals (31.25%) reach complete technical efficiency scores in all five years during 2007-2011 (TE = 1). Another finding extracted from this table is that 15 hospitals (93.75%) are considered as partly efficient units if the value of 0.8 is considered as the cutoff point (Table 2). Other results show that there was a surplus in the studied hospitals' inputs as follows: in the years 2011, 2009 and 2008 only 1 hospital showed input surplus (hospitals No. 6, 1 and 10, respectively), in the year 2010, two units (hospitals number 1 and 12) and in the year 2007, four hospitals had input surplus (No. 1, 6, 7, 14). Table 3 implies that a decrease of 6.42% in the number of active beds (13 beds), 7.04% of nurses (seven nurses), 5.67% of physicians (four physicians) and 4.28% of all other personnel (13 other personnel) throughout all the studied hospitals in the above period may lead to achieve full efficiency (Table 3). The average productivity regarding total factors in the studied hospitals using Malmquist index was 0.983. In addition it was shown that there was an incremental trend in productivity during the studied years with the increase rate of 0.017. The average change in the technical efficiency was reported to be 1.002 and the average rate of technological changes was 0.981. Furthermore, pure average of technical efficiency (managerial) was 1.01 and finally the average of scale efficiency was 0.992 (Table 4). Table 5 shows that the rate of changes in the productivity according to Malmquist index among the studied hospitals was reported between "0.854 to 1.168". Hospital number 2 was presented as the best and hospital number 14 was had the worst performance level considering the rate of productivity change. In addition, the average of total factors' contributing to productivity change among the studied years was less than 1 except the year 2008 that shows an increment in productivity. It is also stated that, the increase was the highest order in 2011 with the average of 0.932 (Table 4).

**Table 2.** Ranking of the Studied Hospitals' Technical Efficiency Applying DEA ^a^

Hospital	2007	2008	2009	2010	2011	Mean ± SD
**5**	1	1	1	1	1	1 ± 0.00
**8**	1	1	1	1	1	1 ± 0.00
**9**	1	1	1	1	1	1 ± 0.00
**13**	1	1	1	1	1	1 ± 0.00
**15**	1	1	1	1	1	1 ± 0.00
**16**	0.939	1	1	1	1	0. ± 0.024
**4**	1	1	1	0.934	1	0. ± .026
**12**	1	1	0.979	0.824	1	0. ± 0.068
**2**	1	1	1	1	0.794	0.959 ± 0.082
**6**	0.946	1	1	1	0.687	0.927± 0.121
**14**	0.642	0.848	0.926	1	1	0.883 ± 0.133
**7**	0.801	0.92	0.922	0.895	0.864	0. ± 0.044
**11**	1	1	0.604	0.79	0.995	0.878 ± 0.158
**10**	1	0.875	0.708	0.831	0.961	0 ± 0.102
**3**	0.951	0.854	0.788	0.756	0.926	0.855 ± 0.075
**1**	0.462	0.745	0.54	0.627	0.552	0.585 ± 0.095
**Mean ± SD**	0 ± 0.156	0.953 ± 0.079	0.904 ± 0.155	0 ± 0.116	0 ± 0.135	0.924 ± 0.105

^a^ Data are presented in Mean ± SD.

**Table 3.** The average of Studied Hospitals’ Input Surplus During 2007-11

Mean	Number of Active Beds	Nurses	Physicians	Other Personnels
**Primary Amounts**	199	96	64	293
**Optimal Amounts**	186	90	61	280
**Surplus**	13	7	4	13
**Surplus, %**	6.42	7.04	5.67	4.28

**Table 4.** Malmquist Index for total Productivity and Total Efficiency During 2008-11 ^a^

Year	Technical Efficiency, Effch	Technological Efficiency, Techch	Managerial Efficiency, Pech	Scale Efficiency, Sech	Total Productivity Changes, Tfpch
**2008**	1.05	0.973	1.06	0.99	1.021
**2009**	0.936	1.062	0.975	0.96	0.994
**2010**	1.022	0.966	1.019	1.003	0.987
**2011**	1.005	0.928	0.989	1.016	0.932
**Mean**	1.002	0.981	1.01	0.992	0.983

^a^ Abbreviations: Effch, technical efficiency change; Techch, technological change; Pech, pure technical efficiency change; Sech, scale efficiency change; Tfpch, total factor productivity change.

**Table 5.** MalmquistIndex for Total Productivity and Total Efficiency in the Hospitals

Hospital	Technical Efficiency Change	Technological Change	Pure Technical Efficiency Change	Scale Efficiency Change	Total Factor Productivity Change
**1**	1.046	0.94	1.193	0.876	0.983
**2**	0.944	0.904	1	0.944	0.854
**3**	0.993	1.013	1	0.993	1.006
**4**	1	0.896	1	1	0.896
**5**	1	0.985	1	1	0.985
**6**	0.923	0.974	0.942	0.98	0.9
**7**	1.019	0.992	1.015	1.004	1.011
**8**	1	0.887	1	1	0.887
**9**	1	1.087	1	1	1.087
**10**	0.99	1.002	1	0.99	0.992
**11**	0.999	1.043	1	0.999	1.042
**12**	1	0.899	1	1	0.899
**13**	1	0.953	1	1	0.953
**14**	1.117	1.046	1.031	1.084	1.168
**15**	1	1.078	1	1	1.078
**16**	1.016	1.028	1	1.016	1.045
**Mean**	1.002	0.981	1.01	0.992	0.983

## 5. Discussion

Being aware of the hospitals’ performance and efficiency is one of the major concerns of health policy makers and health care manager’, although applying a unique scientific technique for hospital performance evaluation, efficiency estimation and determination of the effective factors has not been achieved yet. All the above methods have their own restrictions alongside their strengths, for example despite DEA's advantages in calculating the relative and not absolute efficiency and incorporating multiple input and output factors and also determining optimum level of practice and performance targets (14), there are some noticeable limitations attributed to this method such as eliminating some significant variables and the effect of outliers and missing data (8), where all can lead to wrong estimations of efficiency and performance status (15).

Even though Pabon Lasso gives an instant picture of the hospital, its related indices may be influenced by different factors that cannot be calculated applying this simple model (16). Considering all the above statements, it is inevitable to design multi method studies such as the present one applying different assessment tools to demonstrate a complete and valid picture of the hospitals' efficiency. The present findings show that the average of BOR by the studied hospitals was about 65.91% which is higher than Iran's national average (57.8%) (17).

Moreover, the maximum number of inefficient hospitals in the present study belonged to the years 2007 and 2008 (four hospitals) in contrast with another study on Ahvaz University of Medical Sciences hospitals demonstrating that two hospitals were situated in the first zone in the year 2009 (18). Furthermore, there were only two hospitals in the third part of the present graph which stands for efficiency and optimal level of productivity in the years 2007 and 2009. This number has been increased to three hospitals in 2008 and 2010 and four in 2011 as fully efficient units but Zahiri shows that there were 10 hospitals in the third zone in the year 2009 (18).

These differences can be justified by the fact that the present study only included 16 hospitals in Ahvaz but Zahiri (18) included all the 26 educational and non educational hospitals in khoozestan state that were affiliated to Ahvaz University. Present findings also indicate that the mean score of technical efficiency for the studied hospitals was 0.924 with the minimum of 0.585 for hospital number 1. Furthermore it shows that only 5 hospitals (31.25%) reach complete scale efficiency scores in all five years during 2007-2011 (TE = 1). Fifteen hospitals (93.75%) are considered as partly efficient units considering 0.8 as a cutoff point. Another study performed on Shahid Behshti University of Medical Sciences hospitals showed that the range within the efficient hospitals varied from 0.878 to 0.993 considering 0.8 as an efficiency cut point. The mean efficiency of these hospitals in 2010 was 0.914 in a way that except five hospitals (21.7%) that were fully efficient in technical efficiency, the others (87.3%) could not gain complete technical efficiency scores (TE = 1) (7).

This study like other studies performed in Iran showed that there was a surplus in the studied hospitals’ inputs ( number of personnel, nurses, physicians and beds) in the hospitals affiliated with the medical governmental universities (12). It is obvious that these governmental units do not pay enough attention to the staffing plans because the government pays the staff's salary. A lack of delicate planning for the number of active beds is also recognizable in these hospitals which is not proportional to the population under their coverage.

This finding are justified applying Malmquist index that showed hospital number 2 was presented as the best and hospital number 14 was achieved the worst performance level considering the rate of change in the productivity. So it is recommended to consider the the present results and results of similar studies for the future planning and resource allocation of hospitals. At the same time it is very important to consider need assessment results for each region according to its potentials, population under coverage and other geographical and cultural indices. Furthermore because of potential limitations of each of the above models it is highly recommended to apply different methods of performance evaluation together to reach a complete view of the real status of the hospitals for future planning.
